# Tetra-μ-acetato-bis­{[9-(pyrazin-2-yl)-9*H*-carbazole]copper(II)}

**DOI:** 10.1107/S160053680902710X

**Published:** 2009-07-18

**Authors:** Jin Min Li

**Affiliations:** a School of Chemistry and Chemical Engineering, Shanxi Datong University, Datong 037009, People’s Republic of China

## Abstract

The title complex, [Cu_2_(CH_3_COO)_4_(C_16_H_11_N_3_)_2_], lies on an inversion centre, with four acetate ligands bridging two symmetry-related Cu^II^ ions and two monodentate 9-(pyrazin-2-yl)-9*H*-carbazole ligands coordinating each Cu^II^ ion *via* the N atoms of the pyrazine rings, forming slightly distorted square-pyramidal geometries. There are weak π–π stacking inter­actions between the pyrrole rings of symmetry-related mol­ecules, with a centroid-to-centroid distance of 3.692 (2) Å.

## Related literature

For a related structure, see: Meng *et al.* (2009 ▶).
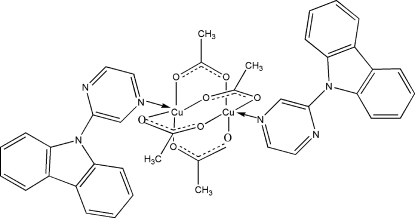


## Experimental

### 

#### Crystal data

[Cu_2_(C_2_H_3_O_2_)_4_(C_16_H_11_N_3_)_2_]
*M* *_r_* = 853.81
Triclinic, 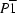

*a* = 8.2608 (12) Å
*b* = 9.7181 (15) Å
*c* = 11.9688 (18) Å
α = 83.002 (2)°
β = 86.756 (2)°
γ = 72.533 (2)°
*V* = 909.5 (2) Å^3^
*Z* = 1
Mo *K*α radiation
μ = 1.23 mm^−1^
*T* = 298 K
0.35 × 0.34 × 0.23 mm

#### Data collection

Bruker SMART APEX CCD diffractometer
Absorption correction: multi-scan (*SADABS*; Sheldrick, 1996 ▶) *T*_min_ = 0.672, *T*_max_ = 0.765
4972 measured reflections
3493 independent reflections
3183 reflections with *I* > 2σ(*I*)
*R*_int_ = 0.018

#### Refinement

*R*[*F* ^2^ > 2σ(*F* ^2^)] = 0.047
*wR*(*F* ^2^) = 0.148
*S* = 1.05
3493 reflections
255 parameters
H-atom parameters constrained
Δρ_max_ = 2.11 e Å^−3^
Δρ_min_ = −0.42 e Å^−3^

### 

Data collection: *SMART* (Bruker, 1997 ▶); cell refinement: *SAINT* (Bruker, 1997 ▶); data reduction: *SAINT*; program(s) used to solve structure: *SHELXTL* (Sheldrick, 2008 ▶); program(s) used to refine structure: *SHELXTL*; molecular graphics: *SHELXTL*; software used to prepare material for publication: *SHELXTL*.

## Supplementary Material

Crystal structure: contains datablocks I, global. DOI: 10.1107/S160053680902710X/lh2859sup1.cif

Structure factors: contains datablocks I. DOI: 10.1107/S160053680902710X/lh2859Isup2.hkl

Additional supplementary materials:  crystallographic information; 3D view; checkCIF report

## Figures and Tables

**Fig. 1.**
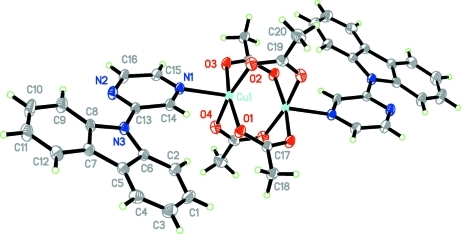
The molecular structure of title complex with the atom-numbering scheme. Displacement ellipsoids are drawn at the 30% probability level.

## supplementary crystallographic information

### Comment

9-(pyrazin-2-yl)-9*H*-carbazole is potentially good ligand for forming complexes with π–π stacking interactions due to the large conjugation plane of carbazole ring system. Interest in synthesizing complexes with π–π stacking interactions motivated us to obtain the title complex and herein the crystal structure of the title compound, (I), is reported.

Figure 1 and Table 1 reveal that the unique Cu^II^ ion is in a slightly distorted square-pyramidal coordination geometry with N1 atom lying at the apex. Four actate anions bridge two symmetry related Cu^II^ ions with a Cu···Cu separation of 2.6120 (7) Å, which is shorter than that of the similar binuclear Cu^II^ complex (Meng *et al.*, 2009). In (I) there is an inversion center in the middle of the two Cu^II^ ions. There is a weak π–π interaction with *Cg*1···*Cg*1^i^ = 3.692 (2) Å (symmetry code: (i): 1-*x*, 1-*y*, 2-*z* and *Cg*1···*Cg*1^i^_perp_ = 3.505 Å; α is 0.00° [*Cg*1 is the centroid of C5–C8/N3 ring; *Cg*1···*Cg*1^i^_perp_ is the perpendicular distance from ring *Cg*1 to ring *Cg*1^i^; α is the dihedral angle between the *Cg*1 ring plane and the *Cg*1^i^ ring plane]. The dihedral angle between pyrazine ring plane and carbazole ring system plane is 20.01 (14)°.

### Experimental

9-(pyrazin-2-yl)-9*H*-carbazole (0.1115 g, 0.455 mmol) was dissolved in 10 ml acetonitrile and copper acetate hydrate (0.0910 g, 0.456 mmol) was added into 10 ml methanol. The solutions were mixed and stirred for a few minutes. Blue single crystals were obtained after the solution had been allowed to stand at room temperature for one month.

### Refinement

All H atoms were placed in calculated positions and refined as riding with C—H = 0.96 Å, *U*_iso_ = 1.5*U*_eq_(C)for methyl group and C—H = 0.93 Å, *U*_iso_ = 1.2*U*_eq_(C) for 9-(pyrazin-2-yl)-9*H*-carbazole H atoms.

### Crystal data

**Table:**

[Cu_2_(C_2_H_3_O_2_)_4_(C_16_H_11_N_3_)_2_]	*Z* = 1
*M**_r_* = 853.81	*F*(000) = 438
Triclinic, *P*1	*D*_x_ = 1.559 Mg m^−^^3^
Hall symbol: -P 1	Mo *K*α radiation, λ = 0.71073 Å
*a* = 8.2608 (12) Å	Cell parameters from 3057 reflections
*b* = 9.7181 (15) Å	θ = 2.6–28.0°
*c* = 11.9688 (18) Å	µ = 1.23 mm^−^^1^
α = 83.002 (2)°	*T* = 298 K
β = 86.756 (2)°	Block, blue
γ = 72.533 (2)°	0.35 × 0.34 × 0.23 mm
*V* = 909.5 (2) Å^3^	

### Data collection

**Table:**

Bruker SMART APEX CCD diffractometer	3493 independent reflections
Radiation source: fine-focus sealed tube	3183 reflections with *I* > 2σ(*I*)
graphite	*R*_int_ = 0.018
φ and ω scans	θ_max_ = 26.0°, θ_min_ = 1.7°
Absorption correction: multi-scan (*SADABS*; Sheldrick, 1996)	*h* = −9→10
*T*_min_ = 0.672, *T*_max_ = 0.765	*k* = −11→11
4972 measured reflections	*l* = −6→14

### Refinement

**Table:**

Refinement on *F*^2^	Primary atom site location: structure-invariant direct methods
Least-squares matrix: full	Secondary atom site location: difference Fourier map
*R*[*F*^2^ > 2σ(*F*^2^)] = 0.047	Hydrogen site location: inferred from neighbouring sites
*wR*(*F*^2^) = 0.148	H-atom parameters constrained
*S* = 1.05	*w* = 1/[σ^2^(*F*_o_^2^) + (0.0986*P*)^2^ + 0.8758*P*] where *P* = (*F*_o_^2^ + 2*F*_c_^2^)/3
3493 reflections	(Δ/σ)_max_ = 0.011
255 parameters	Δρ_max_ = 2.11 e Å^−^^3^
0 restraints	Δρ_min_ = −0.42 e Å^−^^3^

### Special details

**Table:**

Geometry. All e.s.d.'s (except the e.s.d. in the dihedral angle between two l.s. planes) are estimated using the full covariance matrix. The cell e.s.d.'s are taken into account individually in the estimation of e.s.d.'s in distances, angles and torsion angles; correlations between e.s.d.'s in cell parameters are only used when they are defined by crystal symmetry. An approximate (isotropic) treatment of cell e.s.d.'s is used for estimating e.s.d.'s involving l.s. planes.
Refinement. Refinement of *F*^2^ against ALL reflections. The weighted *R*-factor *wR* and goodness of fit *S* are based on *F*^2^, conventional *R*-factors *R* are based on *F*, with *F* set to zero for negative *F*^2^. The threshold expression of *F*^2^ > σ(*F*^2^) is used only for calculating *R*-factors(gt) *etc*. and is not relevant to the choice of reflections for refinement. *R*-factors based on *F*^2^ are statistically about twice as large as those based on *F*, and *R*- factors based on ALL data will be even larger.

### Fractional atomic coordinates and isotropic or equivalent isotropic displacement parameters (Å^2^)

**Table:**

	*x*	*y*	*z*	*U*_iso_*/*U*_eq_	
C1	0.4087 (6)	0.8536 (5)	1.0557 (4)	0.0503 (10)	
H1	0.3442	0.9488	1.0348	0.060*	
C2	0.5243 (5)	0.7790 (4)	0.9784 (3)	0.0406 (8)	
H2	0.5387	0.8235	0.9068	0.049*	
C3	0.3877 (6)	0.7884 (5)	1.1634 (3)	0.0540 (11)	
H3	0.3085	0.8399	1.2131	0.065*	
C4	0.4827 (5)	0.6489 (5)	1.1969 (3)	0.0453 (9)	
H4	0.4690	0.6057	1.2691	0.054*	
C5	0.5990 (4)	0.5734 (4)	1.1220 (3)	0.0334 (7)	
C6	0.6169 (4)	0.6377 (4)	1.0109 (3)	0.0307 (7)	
C7	0.7230 (4)	0.4315 (4)	1.1335 (3)	0.0334 (7)	
C8	0.8123 (4)	0.4117 (4)	1.0305 (3)	0.0322 (7)	
C9	0.9440 (5)	0.2866 (4)	1.0177 (3)	0.0468 (9)	
H9	1.0030	0.2722	0.9495	0.056*	
C10	0.9841 (6)	0.1841 (5)	1.1104 (4)	0.0587 (12)	
H10	1.0721	0.0992	1.1038	0.070*	
C11	0.8984 (6)	0.2028 (5)	1.2130 (4)	0.0545 (11)	
H11	0.9308	0.1321	1.2738	0.065*	
C12	0.7652 (5)	0.3261 (4)	1.2249 (3)	0.0434 (9)	
H12	0.7049	0.3383	1.2928	0.052*	
C13	0.7908 (4)	0.5546 (3)	0.8399 (3)	0.0281 (6)	
C14	0.6825 (4)	0.6558 (4)	0.7633 (3)	0.0326 (7)	
H14	0.5745	0.7071	0.7872	0.039*	
C15	0.8839 (4)	0.6001 (4)	0.6240 (3)	0.0358 (7)	
H15	0.9228	0.6156	0.5504	0.043*	
C16	0.9841 (5)	0.4945 (4)	0.6993 (3)	0.0402 (8)	
H16	1.0871	0.4360	0.6735	0.048*	
C17	0.3340 (4)	1.0792 (3)	0.6772 (3)	0.0296 (7)	
C18	0.2354 (5)	1.1199 (4)	0.7839 (3)	0.0397 (8)	
H18A	0.2835	1.1814	0.8195	0.059*	
H18B	0.2404	1.0337	0.8338	0.059*	
H18C	0.1192	1.1707	0.7663	0.059*	
C19	0.2777 (4)	0.8810 (4)	0.4608 (3)	0.0349 (7)	
C20	0.1397 (5)	0.8131 (5)	0.4428 (4)	0.0529 (10)	
H20A	0.0522	0.8376	0.4996	0.079*	
H20B	0.1872	0.7096	0.4475	0.079*	
H20C	0.0922	0.8491	0.3698	0.079*	
Cu1	0.58853 (4)	0.87807 (4)	0.55418 (3)	0.02722 (17)	
N1	0.7313 (4)	0.6801 (3)	0.6565 (2)	0.0305 (6)	
N2	0.9401 (4)	0.4724 (3)	0.8071 (2)	0.0363 (6)	
N3	0.7439 (4)	0.5373 (3)	0.9538 (2)	0.0315 (6)	
O1	0.4480 (3)	0.9594 (3)	0.6823 (2)	0.0384 (6)	
O2	0.4057 (3)	0.8024 (3)	0.5140 (2)	0.0423 (6)	
O3	0.7047 (3)	0.8328 (3)	0.4096 (2)	0.0385 (6)	
O4	0.7467 (3)	0.9890 (3)	0.5768 (2)	0.0416 (6)	

### Atomic displacement parameters (Å^2^)

**Table:**

	*U*^11^	*U*^22^	*U*^33^	*U*^12^	*U*^13^	*U*^23^
C1	0.055 (2)	0.040 (2)	0.045 (2)	0.0041 (17)	0.0040 (18)	−0.0097 (18)
C2	0.048 (2)	0.0384 (19)	0.0271 (17)	−0.0019 (16)	0.0018 (15)	0.0000 (15)
C3	0.056 (2)	0.056 (3)	0.042 (2)	−0.003 (2)	0.0116 (19)	−0.014 (2)
C4	0.051 (2)	0.055 (2)	0.0289 (18)	−0.0145 (18)	0.0077 (16)	−0.0053 (17)
C5	0.0367 (18)	0.0358 (18)	0.0281 (17)	−0.0130 (14)	−0.0011 (13)	0.0008 (14)
C6	0.0341 (17)	0.0333 (17)	0.0237 (15)	−0.0089 (13)	−0.0003 (12)	−0.0013 (13)
C7	0.0371 (18)	0.0353 (18)	0.0277 (16)	−0.0124 (14)	−0.0032 (13)	0.0031 (14)
C8	0.0355 (17)	0.0332 (17)	0.0243 (16)	−0.0074 (14)	−0.0038 (13)	0.0055 (13)
C9	0.048 (2)	0.043 (2)	0.035 (2)	0.0024 (17)	0.0051 (16)	0.0082 (16)
C10	0.060 (3)	0.040 (2)	0.054 (3)	0.0091 (19)	0.002 (2)	0.0175 (19)
C11	0.066 (3)	0.047 (2)	0.041 (2)	−0.011 (2)	−0.007 (2)	0.0198 (18)
C12	0.054 (2)	0.047 (2)	0.0280 (18)	−0.0169 (18)	−0.0018 (16)	0.0097 (16)
C13	0.0319 (16)	0.0272 (15)	0.0231 (15)	−0.0077 (12)	−0.0012 (12)	0.0025 (12)
C14	0.0315 (16)	0.0341 (17)	0.0253 (15)	−0.0014 (13)	0.0012 (12)	0.0006 (13)
C15	0.0404 (19)	0.0351 (18)	0.0256 (16)	−0.0049 (14)	0.0057 (13)	0.0016 (13)
C16	0.0349 (18)	0.0380 (19)	0.0354 (19)	0.0035 (14)	0.0057 (14)	0.0040 (15)
C17	0.0301 (16)	0.0306 (16)	0.0256 (15)	−0.0061 (13)	0.0037 (12)	−0.0026 (13)
C18	0.0437 (19)	0.0377 (19)	0.0293 (17)	−0.0010 (15)	0.0068 (14)	−0.0038 (15)
C19	0.0340 (17)	0.044 (2)	0.0286 (16)	−0.0138 (15)	0.0053 (13)	−0.0072 (15)
C20	0.044 (2)	0.065 (3)	0.057 (3)	−0.027 (2)	0.0008 (19)	−0.009 (2)
Cu1	0.0280 (3)	0.0261 (3)	0.0220 (2)	−0.00184 (16)	0.00039 (15)	0.00279 (16)
N1	0.0330 (14)	0.0294 (14)	0.0233 (13)	−0.0030 (11)	−0.0007 (11)	0.0039 (11)
N2	0.0341 (15)	0.0350 (15)	0.0309 (15)	−0.0006 (12)	0.0001 (12)	0.0055 (12)
N3	0.0344 (14)	0.0314 (14)	0.0222 (13)	−0.0038 (11)	−0.0008 (11)	0.0068 (11)
O1	0.0408 (13)	0.0348 (13)	0.0285 (12)	0.0027 (10)	0.0064 (10)	0.0000 (10)
O2	0.0409 (14)	0.0401 (14)	0.0466 (15)	−0.0150 (11)	−0.0051 (11)	0.0024 (12)
O3	0.0397 (13)	0.0364 (13)	0.0273 (12)	0.0039 (10)	0.0039 (10)	0.0024 (10)
O4	0.0364 (13)	0.0433 (15)	0.0440 (15)	−0.0121 (11)	−0.0071 (11)	0.0029 (12)

### Geometric parameters (Å, °)

**Table:**

C1—C3	1.391 (6)	C14—H14	0.9300
C1—C2	1.393 (5)	C15—N1	1.332 (4)
C1—H1	0.9300	C15—C16	1.378 (5)
C2—C6	1.376 (5)	C15—H15	0.9300
C2—H2	0.9300	C16—N2	1.330 (4)
C3—C4	1.371 (6)	C16—H16	0.9300
C3—H3	0.9300	C17—O3^i^	1.253 (4)
C4—C5	1.380 (5)	C17—O1	1.256 (4)
C4—H4	0.9300	C17—C18	1.504 (4)
C5—C6	1.419 (5)	C18—H18A	0.9600
C5—C7	1.446 (5)	C18—H18B	0.9600
C6—N3	1.409 (4)	C18—H18C	0.9600
C7—C12	1.386 (5)	C19—O4^i^	1.248 (4)
C7—C8	1.405 (5)	C19—O2	1.257 (4)
C8—C9	1.384 (5)	C19—C20	1.516 (5)
C8—N3	1.423 (4)	C20—H20A	0.9600
C9—C10	1.382 (5)	C20—H20B	0.9600
C9—H9	0.9300	C20—H20C	0.9600
C10—C11	1.385 (6)	Cu1—O3	1.963 (2)
C10—H10	0.9300	Cu1—O1	1.967 (2)
C11—C12	1.378 (6)	Cu1—O2	1.972 (3)
C11—H11	0.9300	Cu1—O4	1.975 (3)
C12—H12	0.9300	Cu1—N1	2.196 (3)
C13—N2	1.321 (4)	Cu1—Cu1^i^	2.6120 (7)
C13—C14	1.398 (4)	O3—C17^i^	1.253 (4)
C13—N3	1.399 (4)	O4—C19^i^	1.248 (4)
C14—N1	1.332 (4)		
			
C3—C1—C2	121.2 (4)	N2—C16—H16	118.5
C3—C1—H1	119.4	C15—C16—H16	118.5
C2—C1—H1	119.4	O3^i^—C17—O1	125.3 (3)
C6—C2—C1	118.3 (3)	O3^i^—C17—C18	117.4 (3)
C6—C2—H2	120.9	O1—C17—C18	117.3 (3)
C1—C2—H2	120.9	C17—C18—H18A	109.5
C4—C3—C1	120.6 (4)	C17—C18—H18B	109.5
C4—C3—H3	119.7	H18A—C18—H18B	109.5
C1—C3—H3	119.7	C17—C18—H18C	109.5
C3—C4—C5	119.1 (4)	H18A—C18—H18C	109.5
C3—C4—H4	120.5	H18B—C18—H18C	109.5
C5—C4—H4	120.5	O4^i^—C19—O2	125.6 (3)
C4—C5—C6	120.5 (3)	O4^i^—C19—C20	117.2 (3)
C4—C5—C7	132.3 (3)	O2—C19—C20	117.2 (3)
C6—C5—C7	107.1 (3)	C19—C20—H20A	109.5
C2—C6—N3	131.4 (3)	C19—C20—H20B	109.5
C2—C6—C5	120.2 (3)	H20A—C20—H20B	109.5
N3—C6—C5	108.3 (3)	C19—C20—H20C	109.5
C12—C7—C8	120.6 (3)	H20A—C20—H20C	109.5
C12—C7—C5	131.4 (4)	H20B—C20—H20C	109.5
C8—C7—C5	107.9 (3)	O3—Cu1—O1	168.81 (10)
C9—C8—C7	120.9 (3)	O3—Cu1—O2	90.06 (12)
C9—C8—N3	131.0 (3)	O1—Cu1—O2	89.26 (12)
C7—C8—N3	108.1 (3)	O3—Cu1—O4	88.60 (11)
C10—C9—C8	117.2 (4)	O1—Cu1—O4	89.90 (12)
C10—C9—H9	121.4	O2—Cu1—O4	168.78 (10)
C8—C9—H9	121.4	O3—Cu1—N1	97.47 (10)
C9—C10—C11	122.6 (4)	O1—Cu1—N1	93.71 (10)
C9—C10—H10	118.7	O2—Cu1—N1	96.33 (11)
C11—C10—H10	118.7	O4—Cu1—N1	94.88 (11)
C12—C11—C10	120.1 (4)	O3—Cu1—Cu1^i^	86.47 (7)
C12—C11—H11	119.9	O1—Cu1—Cu1^i^	82.34 (7)
C10—C11—H11	119.9	O2—Cu1—Cu1^i^	84.81 (8)
C11—C12—C7	118.6 (4)	O4—Cu1—Cu1^i^	83.99 (8)
C11—C12—H12	120.7	N1—Cu1—Cu1^i^	175.89 (7)
C7—C12—H12	120.7	C15—N1—C14	117.8 (3)
N2—C13—C14	121.0 (3)	C15—N1—Cu1	121.7 (2)
N2—C13—N3	118.1 (3)	C14—N1—Cu1	118.9 (2)
C14—C13—N3	120.9 (3)	C13—N2—C16	116.8 (3)
N1—C14—C13	121.1 (3)	C13—N3—C6	126.2 (3)
N1—C14—H14	119.4	C13—N3—C8	125.4 (3)
C13—C14—H14	119.4	C6—N3—C8	108.4 (3)
N1—C15—C16	120.0 (3)	C17—O1—Cu1	125.2 (2)
N1—C15—H15	120.0	C19—O2—Cu1	122.3 (2)
C16—C15—H15	120.0	C17^i^—O3—Cu1	120.6 (2)
N2—C16—C15	123.0 (3)	C19^i^—O4—Cu1	123.3 (2)
			
C3—C1—C2—C6	0.7 (6)	O4—Cu1—N1—C14	97.6 (3)
C2—C1—C3—C4	0.9 (7)	Cu1^i^—Cu1—N1—C14	23.6 (13)
C1—C3—C4—C5	−0.4 (7)	C14—C13—N2—C16	2.4 (5)
C3—C4—C5—C6	−1.7 (6)	N3—C13—N2—C16	−177.8 (3)
C3—C4—C5—C7	175.6 (4)	C15—C16—N2—C13	1.9 (6)
C1—C2—C6—N3	−178.2 (4)	N2—C13—N3—C6	161.2 (3)
C1—C2—C6—C5	−2.8 (6)	C14—C13—N3—C6	−19.0 (5)
C4—C5—C6—C2	3.4 (5)	N2—C13—N3—C8	−21.7 (5)
C7—C5—C6—C2	−174.5 (3)	C14—C13—N3—C8	158.2 (3)
C4—C5—C6—N3	179.8 (3)	C2—C6—N3—C13	−9.3 (6)
C7—C5—C6—N3	1.9 (4)	C5—C6—N3—C13	174.8 (3)
C4—C5—C7—C12	−0.6 (7)	C2—C6—N3—C8	173.2 (4)
C6—C5—C7—C12	176.9 (4)	C5—C6—N3—C8	−2.7 (4)
C4—C5—C7—C8	−177.9 (4)	C9—C8—N3—C13	6.0 (6)
C6—C5—C7—C8	−0.3 (4)	C7—C8—N3—C13	−175.1 (3)
C12—C7—C8—C9	0.2 (6)	C9—C8—N3—C6	−176.5 (4)
C5—C7—C8—C9	177.8 (3)	C7—C8—N3—C6	2.5 (4)
C12—C7—C8—N3	−178.9 (3)	O3^i^—C17—O1—Cu1	−2.1 (5)
C5—C7—C8—N3	−1.3 (4)	C18—C17—O1—Cu1	177.6 (2)
C7—C8—C9—C10	−0.7 (6)	O3—Cu1—O1—C17	2.9 (7)
N3—C8—C9—C10	178.1 (4)	O2—Cu1—O1—C17	−83.6 (3)
C8—C9—C10—C11	0.0 (8)	O4—Cu1—O1—C17	85.2 (3)
C9—C10—C11—C12	1.3 (8)	N1—Cu1—O1—C17	−179.9 (3)
C10—C11—C12—C7	−1.8 (7)	Cu1^i^—Cu1—O1—C17	1.2 (3)
C8—C7—C12—C11	1.1 (6)	O4^i^—C19—O2—Cu1	3.3 (5)
C5—C7—C12—C11	−175.9 (4)	C20—C19—O2—Cu1	−175.5 (2)
N2—C13—C14—N1	−5.0 (5)	O3—Cu1—O2—C19	−88.5 (3)
N3—C13—C14—N1	175.2 (3)	O1—Cu1—O2—C19	80.3 (3)
N1—C15—C16—N2	−3.9 (6)	O4—Cu1—O2—C19	−5.4 (7)
C16—C15—N1—C14	1.2 (5)	N1—Cu1—O2—C19	173.9 (3)
C16—C15—N1—Cu1	166.9 (3)	Cu1^i^—Cu1—O2—C19	−2.1 (3)
C13—C14—N1—C15	3.0 (5)	O1—Cu1—O3—C17^i^	−1.3 (7)
C13—C14—N1—Cu1	−163.1 (3)	O2—Cu1—O3—C17^i^	85.2 (3)
O3—Cu1—N1—C15	21.3 (3)	O4—Cu1—O3—C17^i^	−83.7 (3)
O1—Cu1—N1—C15	−158.1 (3)	N1—Cu1—O3—C17^i^	−178.4 (3)
O2—Cu1—N1—C15	112.2 (3)	Cu1^i^—Cu1—O3—C17^i^	0.4 (3)
O4—Cu1—N1—C15	−67.9 (3)	O3—Cu1—O4—C19^i^	86.1 (3)
Cu1^i^—Cu1—N1—C15	−141.9 (10)	O1—Cu1—O4—C19^i^	−82.8 (3)
O3—Cu1—N1—C14	−173.2 (3)	O2—Cu1—O4—C19^i^	2.8 (7)
O1—Cu1—N1—C14	7.4 (3)	N1—Cu1—O4—C19^i^	−176.6 (3)
O2—Cu1—N1—C14	−82.3 (3)	Cu1^i^—Cu1—O4—C19^i^	−0.5 (3)

Symmetry codes: (i) −*x*+1, −*y*+2, −*z*+1.
